# Site of cochlear stimulation and its effect on electrically evoked compound action potentials using the MED-EL standard electrode array

**DOI:** 10.1186/1475-925X-8-40

**Published:** 2009-12-16

**Authors:** Stefan Brill, Joachim Müller, Rudolf Hagen, Alexander Möltner, Steffi-Johanna Brockmeier, Thomas Stark, Silke Helbig, Jan Maurer, Thomas Zahnert, Clemens Zierhofer, Peter Nopp, Ilona Anderson, Stefan Strahl

**Affiliations:** 1 Department of Otorhinolaryngology, Plastic, Aesthetic and Reconstructive Head and Neck Surgery, University of Wuerzburg, Würzburg, Germany; 2 HNO Klinik, Klinikum rechts der Isar der TU München, München, Germany; 3 St Elisabeth Hospital, HNO-Klinik der Ruhr Universität, Bochum, Germany; 4 HNO-Heilkunde, Klinikum der JW Goethe-Universität, Frankfurt/Main, Germany; 5 HNO-Klinik, Katholisches Klinikum Koblenz, Koblenz, Germany; 6 HNO- Heilkunde, Universitätsklinikum Carl Gustav Carus, Dresden, Germany; 7 HNO Klinik der Universität Basel, Basel, Switzerland; 8 Christian-Doppler-Labor, Universität Innsbruck, Innsbruck, Austria; 9 MED-EL GmbH, Innsbruck, Austria

## Abstract

**Background:**

The standard electrode array for the MED-EL MAESTRO cochlear implant system is 31 mm in length which allows an insertion angle of approximately 720°. When fully inserted, this long electrode array is capable of stimulating the most apical region of the cochlea. No investigation has explored Electrically Evoked Compound Action Potential (ECAP) recordings in this region with a large number of subjects using a commercially available cochlear implant system. The aim of this study is to determine if certain properties of ECAP recordings vary, depending on the stimulation site in the cochlea.

**Methods:**

Recordings of auditory nerve responses were conducted in 67 subjects to demonstrate the feasibility of ECAP recordings using the Auditory Nerve Response Telemetry (ART™) feature of the MED-EL MAESTRO system software. These recordings were then analyzed based on the site of cochlear stimulation defined as basal, middle and apical to determine if the amplitude, threshold and slope of the amplitude growth function and the refractory time differs depending on the region of stimulation.

**Results:**

Findings show significant differences in the ECAP recordings depending on the stimulation site. Comparing the apical with the basal region, on average higher amplitudes, lower thresholds and steeper slopes of the amplitude growth function have been observed. The refractory time shows an overall dependence on cochlear region; however post-hoc tests showed no significant effect between individual regions.

**Conclusions:**

Obtaining ECAP recordings is also possible in the most apical region of the cochlea. However, differences can be observed depending on the region of the cochlea stimulated. Specifically, significant higher ECAP amplitude, lower thresholds and steeper amplitude growth function slopes have been observed in the apical region. These differences could be explained by the location of the stimulating electrode with respect to the neural tissue in the cochlea, a higher density, or an increased neural survival rate of neural tissue in the apex.

**Trial registration:**

The Clinical Investigation has the Competent Authority registration number DE/CA126/AP4/3332/18/05.

## Background

Objective measures are a widely used and valuable tool in the field of cochlear implants (CIs). During surgery they provide first indications of successful implantation and after surgery they are used to facilitate the individual fitting of stimulation parameters. One objective measure of the auditory nerve's response to stimulation is the Electrically Evoked Compound Action Potential (ECAP). This response is particularly advantageous because it allows the clinician to directly measure auditory nerve fibre potentials on implanted patients. In analyzing the physiological response to the electrical stimulation transmitted by the implant, information can be obtained regarding the expected and actual function of the peripheral nerve. This information can be used intraoperatively to adjust the placement of the intracochlear electrode and for technical functional testing. Postoperatively, the ECAP recordings can be used to measure the neuronal potentials elicited by electrode stimulation along the basilar membrane. In addition, these measurements may help to determine the upper and lower limits of the stimulation current [the hearing threshold (THR) and the maximum comfort level (MCL)] in cases of fitting [1-7] or to evaluate the stimulation current field along the cochlea and the interaction of individual electrodes [8-10].

Stimulation of an intracochlear electrode results in the excitation of specific populations of neural fibres that are distributed along the basilar membrane. Previous investigations have been limited in terms of how much of the cochlea can be analyzed because of maximal insertion depths and insertion angles of the electrode arrays. For this investigation, the Auditory Nerve Telemetry (ART) [11] in the MED-EL MAESTRO CI system (MED-EL, Innsbruck, Austria) was used to perform ECAP recordings. The MED-EL standard electrode array allows an insertion depth of 31 mm or 720° and comprises 12 channels [12], allowing access to the most apical region of the cochlea. The channels are spaced 2.4 mm apart, and are numbered E 1 to E 12, from apical to basal.

The primary objective of this study was to establish whether a difference exists in ECAP properties depending on the region of the cochlea that is stimulated and measured. Previous studies using other CI systems found increased ECAP amplitudes at the apical electrodes [13,14]. Note that these publications did not realize a full cochlear coverage. Also electrophysiological models did not analyze a (deep) apical stimulation, comparing it with a middle or basal stimulation. Therefore in this study, an analysis of amplitude, threshold, and slope of the ECAP recordings obtained in various regions of cochlear stimulation was conducted. A secondary objective of this study was to determine if the ECAP amplitude in relative refractory state shows systematic differences for different stimulation and recording sites.

## Methods

### Subjects

46 experienced and 21 inexperienced CI users were recruited from 13 centres in Germany for a clinical study [15,16] investigating CI coding strategies and ECAP recordings. The study was approved by the International Freiburger Ethic Commission (FECI 05/2134). Of these 67 CI subjects, 34 were women and 33 were men. The mean age at onset of hearing loss was 43.4 years with a range of 1 to 73 years. The mean age at implantation was 55.4 years with a range of 20 to 76 years. 37 subjects were implanted on the left side and 30 were implanted on the right. All subjects were postlingually deaf, (defined as an onset of severe-to-profound sensorineural hearing loss occurring after 6 years of age) and were unilaterally implanted with a MED-EL PULSARci^100 ^or SONATAti^100 ^using the standard electrode array. All subjects were required to have at least 10 active electrodes at the last fitting. All subjects were native German speakers.

### Subject groups

Experienced subjects were adults (18 years or older) with a minimum of six months of device experience (mean = 1.4 years; range = 7 to 31 months). The inexperienced subjects were adults who received their first CI after having undergone a hearing aid trial where minimal aided benefit from hearing aid(s) was determined. These subjects fell within the medical and audiological guidelines established by their respective centres for cochlear implantation. Apart from experience with the device, the inclusion criteria were the same for both the experienced and inexperienced groups.

### Statistical Analysis

If for one subject more than one recording of an ECAP property was available, the mean value was used in the analysis. To test the hypotheses, a general linear model was applied and analyses of variance (ANOVA) for repeated measurements [17] with the region as factor were performed for each test condition. If the assumption of sphericity was not tenable according to Mauchly's test [18], the Greenhouse-Geisser correction [19] was applied. To detect significant effects of the region on the ECAP measurement, parametric paired Student's t-tests were used. After adjustments for multiple comparisons with the use of Bonferroni's procedure [20] p-values of less than 0.017 were considered to indicate statistical significance. The software employed for the statistical analysis was Matlab Rev 7.0.0.19920 with the Statistics Toolbox Version 5.0.

### ECAP Measurements

ECAP measurements were performed as part of a study [15,16] evaluating ECAP recordings, and the performance of subjects with the Fine Structure Processing (FSP) strategy as improved in the OPUS audio processors, acutely and after three months of device use. ECAPs were recorded postoperatively using the MAESTRO 2.0 system software connected to a DIB II interface box. For the experienced subjects ECAP recordings were obtained at acute switch-over from their clinical TEMPO+ speech processor to the OPUS 1 speech processor employing the FSP coding strategy, and for the inexperienced subjects at initial stimulation. If no ECAP could be recorded from a subject of either group, measurements were reattempted three months after the initial test date.

**Figure 1:**
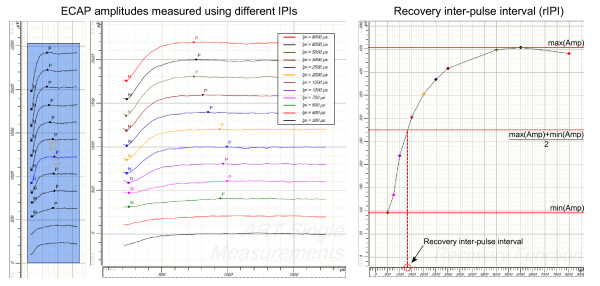
**Recovery inter-pulse interval (rIPI)**. To compute the recovery inter-pulse interval (rIPI) the ECAP amplitude is measured for a range of different IPIs. The rIPI is defined as the IPI that would result in the mean of the maximal and the minimal amplitude measured.

ECAP amplitude, threshold and slope were measured using amplitude growth sequences. These recording sequences consisted of several single recordings that had one stimulation pulse followed by an ECAP measurement. The single recordings were separated by approximately 30 ms, assuming a relative refractory time below 10 ms. The phase duration was set to 30 μs and the stimulation amplitude was defined in current unit (cu), where 1 cu corresponds to approximately 1 μA. Across the sequence, the stimulation amplitude for a single recording was raised from 0 cu to a maximum value, which typically correlates to the patient's MCL, for example, 640 cu. Additionally, in the experienced users, recovery sequence results were measured. Recovery sequences consisted of independent single recordings that had two stimulation pulses followed by an ECAP measurement. The time between the onset of the two pulses (inter-pulse interval or IPI) was 300, 400, 500, 750, 1000, 1500, 2000, 2500, 3000, 5000, 6000 and 8000 μs. The recovery inter-pulse interval (rIPI) was defined as that IPI that would result in an ECAP having an amplitude that is the mean of the maximal and the minimal amplitude measured in the recovery sequence (see Figure 1). The fixed sampling of the IPIs defines the maximal and minimal amplitude, i.e. the rIPI, independent of any user interpretation and allows for a relative comparison between the different electrode positions. An alternative way to reduce the influence of any measurement noise or residual artifact would be an extrapolation of the recovery function [21], which was not possible as the report forms of the analyzed study only contained the minimal and maximal amplitude and the rIPI itself.

For all measurements, the stimulation artifact was removed using an alternating stimulation approach. Each measurement is performed twice, with a cathodic/anodic and an anodic/cathodic stimulation pulse, respectively. When averaging the two measurements, the stimulation artifact vanishes and the ECAP signal remains. The recording artifact was removed subtracting a zero amplitude template [2].

### Electrode Regions

The stimulation region was subdivided into three regions according to the electrode and its approximate location within the cochlea (see Table 1). In subjects with 12 active electrodes, the basal region was defined as Electrodes 9 to 12, the middle region included Electrodes 5 to 8 and the apical region included Electrodes 1 to 4. If the most basal electrode (Electrode 12) was deactivated, it was assumed that the electrode array was not fully inserted and the mapping was shifted by one electrode, resulting in the apical region containing Electrodes 1 to 3 (instead of Electrodes 1 to 4). Likewise, if Electrodes 11 and 12 were not activated, the ranges shifted by two electrodes so that the apical region included Electrodes 1 and 2 only. If any other electrodes were not activated, no shifts were applied and the deactivated electrode was excluded from the data analysis. Of the 67 subjects, all 12 electrodes were activated in 48 users (71.6%). 11 electrodes were activated in 14 users (20.9%) and for 5 users (7.5%), 10 electrodes were activated.

**Table 1:** Mapping of the electrodes to the cochlear region depending on the electrode insertion depth.

Electrode	1	2	3	4	5	6	7	8	9	10	11	12
Active Electrodes: 1-12	a	a	a	a	m	m	m	m	b	b	b	b
Active Electrodes: 1-11	a	a	a	m	m	m	m	b	b	b	b	-
Active Electrodes: 1-10	a	a	m	m	m	m	b	b	b	b	-	-

Regions are encoded as: a = apical, m = middle, b = basal.

## Results

The ECAP results reported below are summarized in Table 2. Results from the statistical tests are summarized in Table 3. The data collected was subsequently analyzed for the purpose of determining if differences existed in ECAP recordings obtained in various regions of the cochlea. In this analysis, the experienced and inexperienced group were merged into one group (N = 67) in order to gain a more consolidated analysis of possible effects depending of the stimulation site. To rule out any effects of a significant difference between the experienced and inexperienced groups, we performed t-tests for amplitude, threshold and slope. The results revealed no significant differences between the two groups.

**Table 2:** The ECAP measurement results reported as mean values with the standard deviation.

	basal	middle	apical	full region
**ECAP amplitude**	220.8 ± 114.4 μV	257.9 ± 129.7 μV	341.3 ± 200.5 μV	273.3 ± 159.6 μV
**ECAP threshold**	356.2 ± 114.0 cu	337.8 ± 100.2 cu	307.3 ± 113.7 cu	333.8 ± 110.3 cu
**ECAP amplitude growth function**	0.747 ± 0.389 μV/cu	0.766 ± 0.384 μV/cu	1.092 ± 0.638 μV/cu	0.869 ± 0.506 μV/cu
**ECAP amplitude recovery sequence**	163.3 ± 87.7 μV	192.8 ± 70.2 μV	223.4 ± 104.2 μV	193.2 ± 90.1 μV
**ECAP rIPI**	1267.9 ± 301.9 μs	1236.0 ± 415.6 μs	1519.0 ± 431.8 μs	1341.0 ± 400.1 μs

**Table 3:** Observed regional effects on the respective ECAP features.

	ANOVA	t-test basal-apical	t-test middle-apical	t-test middle-basal	Mauchly's Test	Greenhouse Geisser
**ECAP amplitude**	**P < 0.001** **F = 10.994**	**p = 0.0007**	**p = 0.0035**	p = 0.0527	No sphericity	ε = 0.748
**ECAP threshold**	**P = 0.003** **F = 5.376**	**p = 0.0026**	p = 0.0511	p = 0.2314	No sphericity	ε = 0.999
**ECAP slope (ampl. growth function)**	**P < 0.001** **F = 12.038**	**p = 0.0014**	**p = 0.0002**	p = 0.7055	No sphericity	ε = 0.690
**ECAP amplitude (recovery function)**	**P = 0.045** **F = 3.460**	**p = 0.0086**	p = 0.2770	p = 0.1775	sphericity	-
**ECAP Recovery** **Interpulse nterval (rIPI)**	**P = 0.029** **F = 4.015**	p = 0.0590	p = 0.0262	p = 0.7198	sphericity	-

The results of the statistical analysis are shown. Significant results are printed in boldface. The Bonferroni corrected significance level was α = 0.0167.

### Ecap Amplitude Growth Sequence

ECAP measurements were possible for 58 users accounting for 86.6% of all subjects. Within our subjects, the presence or absence of ECAP recordings varied between ECAP recordings sites. The presence of a clear response was greatest when the stimulating electrode and measuring electrode were in the middle and apical region of the cochlea. Recordings from the middle region were obtained in 52 (77.6%) subjects, and from the apical region in 51 (76.1%). In the basal region, responses were obtained in 38 subjects (56.7%). The small number of subjects who had responses in the basal region reduced the number of subjects for whom ECAPs were successfully measured in all three regions to 34 users (50.7%). Data reporting is therefore based on those subjects (N = 34) except where otherwise stated.

### Ecap Recovery Sequence

Recovery sequences were measured in the experienced group (N = 46). Unlike for the amplitude growth sequence, variability in the presence or absence of a clear response depending on the cochlear region was not seen. Clear responses were recorded for 21 subjects (45.7%) in the basal region, for 25 subjects (54.3%) in the middle region, and for 21 subjects (45.7%) in the apical region. There were 16 subjects (34.8%) for whom an ECAP recovery sequence could be measured in all three regions and 29 subjects (63.0%) for whom an ECAP recovery sequence determination was possible in any region.

### Ecap Amplitude Using Amplitude Growth Sequence

**Figure 2:**
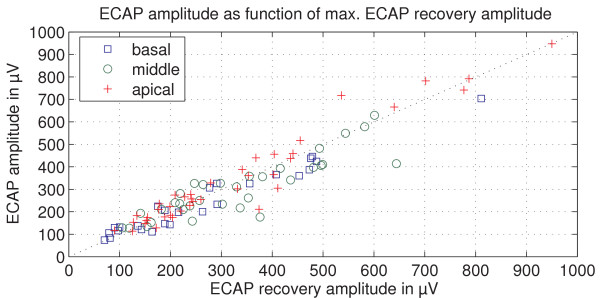
**ECAP amplitude as function of maximal ECAP recovery amplitude**. The maximal amplitude measured with the ECAP recovery sequence for the group of experienced users is plotted against the measured ECAP amplitudes. The Pearson's correlation coefficient is r = 0.945.

ECAP amplitude recordings using an amplitude growth sequence were compared to maximum ECAP amplitudes from ECAP recovery sequences. They showed a strong correlation (Pearson's coefficient, r = 0.945) (Figure 2) and a t-test showed no significant differences between them (p = 0.116). This confirms the correctness of the ECAP amplitude measurements in the study.

**Figure 3:**
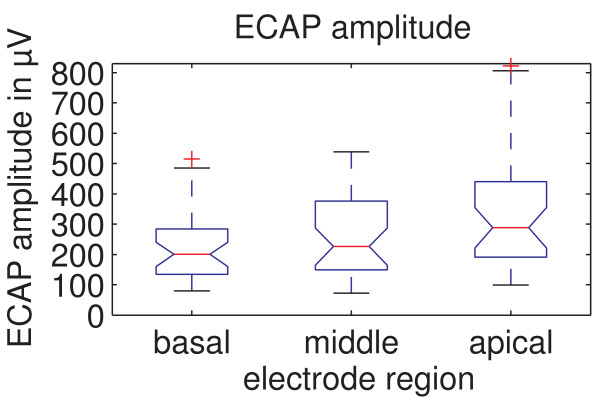
**ECAP amplitudes for the different regions of the cochlea**. ECAP amplitudes measured are shown for the different regions. There are significant differences between the basal and apical regions (p < 0.001) as well as the middle and apical regions (p = 0.003).

In Figure 3 ECAP amplitudes measured with an amplitude growth sequence are shown for the different cochlear stimulation sites. The mean ECAP amplitude was the lowest for the basal region (220.8 ± 114.4 μV); compared to 257.9 ± 129.7 μV for the middle and 341.3 ± 200.5 μV for the apical region. Mauchly's sphericity test, which was always performed with a Bonferroni corrected significance level of α = 0.05/3, showed that the assumption of sphericity is violated. Therefore, a Greenhouse-Geisser correction of ε = 0.748 was applied to the ANOVA resulting in p < 0.001 and F = 10.994. These results show that there is a significant effect of the stimulation region on the ECAP amplitude. Paired t-tests revealed that significant differences on ECAP amplitude exist between the basal and apical regions (p < 0.001) as well as the middle and apical regions (p = 0.004), though between the basal and middle regions no significant effect was found (p = 0.05).

### Ecap Threshold Using Amplitude Growth Sequence

**Figure 4:**
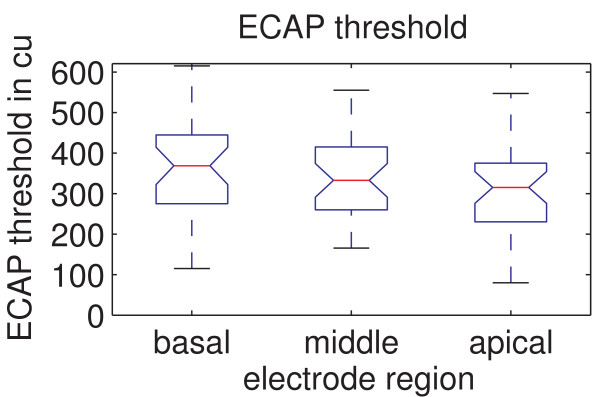
**ECAP thresholds for the different regions**. The measured ECAP thresholds shown for the different regions. There is a significant difference between the basal and apical region (p = 0.0026).

In Figure 4 the ECAP thresholds analyzed for all three regions are shown. The mean ECAP threshold was with 356.2 ± 114.0 cu the highest for the basal region, compared to 337.8 ± 100.2 cu for the middle region, and 307.3 ± 113.7 cu for the apical region. A Greenhouse-Geisser correction of ε = 0.999 was applied to the ANOVA as Mauchly's sphericity test showed that the assumption of sphericity is violated. The ANOVA revealed a significant effect of stimulation region on the ECAP threshold (p = 0.007 and F = 5.376). Post-hoc testing further showed that a significant difference in the ECAP threshold exists between the basal and apical regions (p = 0.003). The basal and middle regions (p = 0.231) as well as the middle and apical regions (p = 0.05) did not show significant differences.

### Slope of Ecap Amplitude Growth Function

**Figure 5:**
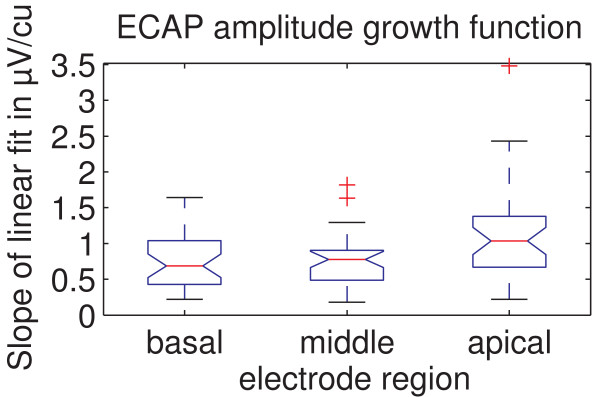
**ECAP amplitude growth function for the different regions**. The slope of the fitted ECAP amplitude growth function is shown for the different regions. There are significant differences between the basal and apical region (p = 0.001) as well as the middle and apical region (p < 0.001).

The results shown in Figure 5 represent the slope of a linear fit applied to the rising part of the ECAP amplitude growth function. This linear fit is by default applied automatically by the MAESTRO clinical software. The smallest inclination of the mean slope was observed for the basal region (0.747 ± 0.389 μV/cu), compared to 0.766 ± 0.384 μV/cu for the middle region and 1.092 ± 0.638 μV/cu for the apical region. Mauchly's sphericity test showed that the assumption of sphericity is violated for this data set. An ANOVA with a Greenhouse-Geisser correction of ε = 0.690 showed a significant effect of the three different stimulation regions on the ECAP growth function (p < 0.001 and F = 12.038). Post-hoc tests revealed that there is a significant effect between the basal and apical regions (p = 0.001) as well as between the middle and apical regions (p < 0.001). The basal and middle regions showed no significant difference (p = 0.706).

### Ecap Amplitude Derived Using Recovery Sequence

**Figure 6:**
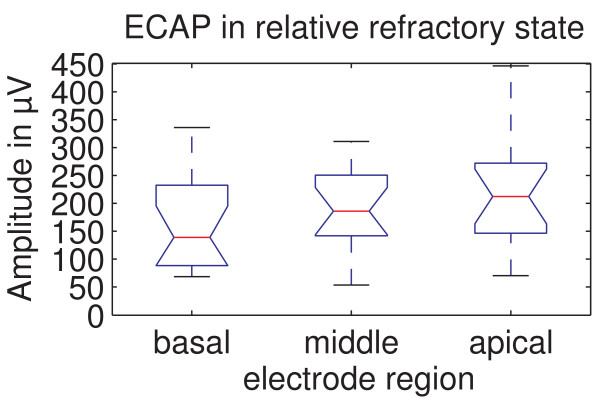
**ECAP amplitude in relative refractory state for the different regions**. The ECAP amplitude in relative refractory state shown for the different regions. There is a significant difference between the basal and apical region (p = 0.008).

The results shown in Figure 6 represent the ECAP amplitude at the recovery inter-pulse interval (rIPI) as defined in Figure 1. The ECAP amplitudes presented here thus represent ECAP amplitudes in relative refractory state. They were derived using a recovery sequence for 16 experienced subjects where ECAP recovery sequences were available for all 3 regions. The mean ECAP amplitude in the basal region was 163.3 ± 87.7 μV, 192.8 ± 70.2 μV in the middle region and 223.4 ± 104.2 μV in the apical region. Mauchly's sphericity test showed that the assumption of sphericity is tenable. The ANOVA yielded p = 0.045 and F = 3.460, indicating a significant effect of stimulation region on ECAP amplitude in relative refractory state. Post-hoc tests revealed a significant effect between the basal and apical regions (p = 0.008). No significant effect was found between the middle and apical regions (p = 0.277) as well as the basal and middle regions (p = 0.178).

### Recovery Inter-Pulse Interval (rIPI)

**Figure 7:**
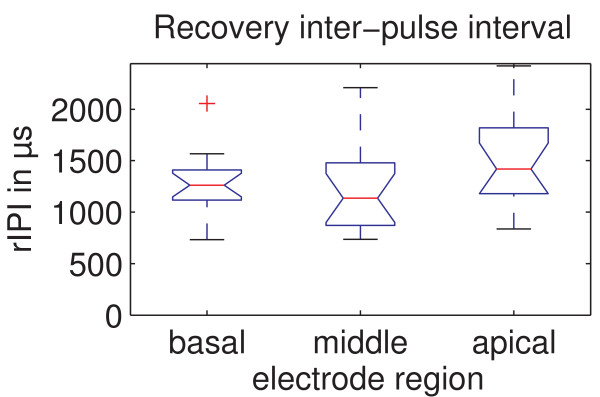
**Recovery inter-pulse interval (rIPI) for the different regions**. The recovery inter-pulse interval (rIPI, as defined in Figure 1) shows an overall dependence on cochlear region, however post-hoc tests showed no significant effect between individual regions.

The recovery inter-pulse intervals as defined in Figure 1 are presented in Figure 7. The mean recovery inter-pulse interval was 1267.9 ± 301.9 μs for the basal region, 1236.0 ± 415.6 μs for the middle region and 1519.0 ± 431.8 μs for the apical region for the 16 experienced subjects where ECAP recovery sequences were available for all 3 regions. Mauchly's sphericity test with a Bonferroni corrected significance level of α = 0.05/3 showed that the assumption of sphericity is tenable. The ANOVA showed with p = 0.029 and F = 4.015 that there is a significant effect of stimulation region on recovery inter-pulse interval. However, post-hoc tests revealed no significant effects between the basal and apical regions (p = 0.059), middle and apical regions (p = 0.026) or the basal and middle regions (p = 0.719).

## Discussion

In this study we examined if certain properties of ECAP recordings vary, depending on the stimulation site in the cochlea. To various degrees, we found a significant effect of stimulation site on ECAP amplitude, ECAP threshold, slope of ECAP amplitude growth function and ECAP recovery inter-pulse interval (Table 2, Table 3).

The significant increase in ECAP amplitude towards the apical region could be explained by a narrowed distance between the recording electrode and the stimulated neural tissue (because of reduced diameter of the cochlear turns towards the apex). Another possible factor is the neural tissue itself. A greater density or survival rate of neuronal tissue adjacent to the electrode in the apical region could also explain the increase in ECAP amplitude which is also supported by the steeper growth function observable in Figure 5. Better neural survival of neural structures in the apex might be produced by later deafening of the apex, e.g. in many cases of progredient deafness. Differences in impedances of the electrode-tissue interfaces between cochlear regions can not explain this effect because the implant uses current sources and high-impedance recording circuitry and is therefore not affected by different electrode-tissue interface impedances.

We also investigated for the analyzed subjects whether an increased stimulation amplitude towards the apical region might be responsible for the amplitude increase. For the basal region the mean stimulation amplitude was 654.0 ± 224.4 μV. For the middle and apical region, mean stimulation amplitudes were 723.1 ± 293.8 μV and 706.9 ± 317.1 μV, respectively. An ANOVA showed that the stimulus amplitudes are not dependent of the measurement region (p = 0.244, F = 1.445). As stimulation amplitudes at initial stimulation usually differ from amplitudes for experienced users, we did also an ANOVA for the experienced users only, which in contrast to the data above showed a region dependency of the stimulation amplitudes (p = 0.001, F = 7.730). Paired t-tests show that there is a significant difference between the basal and middle (p = 0.001) as well as the basal and apical region (p = 0.004). This could partially have contributed to the ECAP amplitude increase towards the apex. The middle and apical region, however, shows no significant difference (p = 0.812) in stimulation amplitude although a significant difference in ECAP amplitude was found here also (p = 0.004), as mentioned above. Therefore the chosen stimulation amplitude can only partly explain the region dependency of the ECAP amplitude. This is confirmed by the fact that studies using other CI systems also found increased ECAP amplitudes for more apical electrodes [13,14].

The significant decrease in ECAP threshold towards the apex could again be attributed to the narrowed distance between the recording electrode and the surrounding tissue. Additionally, similar to above, a greater density of neuronal tissue or survival rate adjacent to the electrode could also lead to a reduction in the stimulation amplitude required to trigger an action potential. As mentioned previously, regional differences in the impedance of the electrodes cannot explain the decrease towards the apex.

The slope of the ECAP growth function should strongly correlate with an increase in the number of neurons that respond to every increment in stimulation level. The steeper growth function shown in Figure 5 indicates that towards the apical region, a greater number of neural elements are activated for every increment in stimulation level. Since spiral ganglion cells do not extend into the apical region of the cochlea, these neural elements should mainly be afferent peripheral axons.

As the ECAP amplitudes from the recovery sequences did not differ significantly from those from growth sequences, ECAP amplitudes from recovery sequences also showed a region dependency effect. The significant difference in ECAP amplitudes between the basal and apical region is also found here. The fact that - in contrast to ECAP amplitudes from growth sequences - no significant difference between the middle and apical region could be found here is presumably due to the smaller subject group (16 instead of 34) in which recovery sequences were performed.

The recovery interpulse interval (rIPI) shows an overall effect but no significant post-hoc differences for different stimulation and recording sites. There are at least two different mechanisms that could lead to changes in the latency of ECAP potentials along the cochlea: Some firing features of type II spiral ganglion neurons are cochlear region dependent; the latency is reported to be longer for the apical region [22,23] (determined in murine cochlea). Secondly, the latency depends on the site of stimulation and recording sites. These locations depend on the spiral ganglion cell arrangement between apex and base that is reported to be different [24,25] (human cochlea). This effect is also assumed to be the reason for the double P peaks, seen in 9.5% of ECAP responses [26].

The observed increase in ECAP amplitude towards the apex of the cochlea adds another aspect to the discussion about complete electrode insertion in cochlear implantation. We interpret this result as further evidence for the usefulness of apical cochlear stimulation. The data from objective measurements presented here complement data from behavioural assessment which show that speech discrimination improves when the most apical region of the cochlear is stimulated along with the medial and basal region [27,12]. Restricting the most apical stimulation to 300° to 400° insertion angle does not take advantage of this portion of the cochlea.

## Conclusions

Four different properties of the ECAP in adult subjects were analysed based on the region of the cochlea where the response was generated. All four properties analysed were found to be affected by the cochlear region. Apical recordings showed on average higher ECAP amplitudes, lower ECAP thresholds, and steeper slopes of the ECAP amplitude growth function. Also the ECAP refractory time showed a significant effect of stimulation region. These regional differences could be due to the closer proximity of the stimulating electrode to neural tissue in the apex and/or to a higher density or survival rate of neural tissue in the apex, which could also explain the robustness of the apical response and the steeper growth function. The recovery inter-pulse interval showed an overall dependence on cochlear region while significant effects between the individual regions could not be shown.

The novel available large number of ECAP measurements from the most apical region of the cochlea show that significant differences exist between the apical and the basal region. The apical ECAP recordings show a clear response with higher (on average) amplitudes and a lower threshold both of which are advantageous in terms of measurement success. These findings imply that future studies being conducted on ECAPs should include, wherever possible, a regional analysis of the results to incorporate any region dependent effects.

## Competing interests

The study was sponsored by MED-EL GmbH.

## Authors' contributions

SB, JM, RH, AM, S-JB, TS, SH, MD, JM, TZ carried out the clinical trials. CZ was responsible for the ECAP-signal processing concept and for the system implementation. PN and IA conceived the study, undertook its design and helped to draft the manuscript. SS performed further statistical analyses and wrote the manuscript. All authors read and approved the final manuscript.
